# Whole-body voxel-based internal dosimetry using deep learning

**DOI:** 10.1007/s00259-020-05013-4

**Published:** 2020-09-01

**Authors:** Azadeh Akhavanallaf, Iscaac Shiri, Hossein Arabi, Habib Zaidi

**Affiliations:** 1 Division of Nuclear Medicine and Molecular Imaging, Geneva University Hospital, CH-1211 Geneva, Switzerland; 2 Geneva University Neurocenter, Geneva University, CH-1205 Geneva, Switzerland; 3 Department of Nuclear Medicine and Molecular Imaging, University Medical Center Groningen, University of Groningen, 9700 RB Groningen, Netherlands; 4 Department of Nuclear Medicine, University of Southern Denmark, DK-500 Odense, Denmark

**Keywords:** Internal dosimetry, Patient specific, Monte Carlo, Deep learning, Voxel based

## Abstract

**Purpose:**

In the era of precision medicine, patient-specific dose calculation using Monte Carlo (MC) simulations is deemed the gold standard technique for risk-benefit analysis of radiation hazards and correlation with patient outcome. Hence, we propose a novel method to perform whole-body personalized organ-level dosimetry taking into account the heterogeneity of activity distribution, non-uniformity of surrounding medium, and patient-specific anatomy using deep learning algorithms.

**Methods:**

We extended the voxel-scale MIRD approach from single S-value kernel to specific S-value kernels corresponding to patient-specific anatomy to construct 3D dose maps using hybrid emission/transmission image sets. In this context, we employed a Deep Neural Network (DNN) to predict the distribution of deposited energy, representing specific S-values, from a single source in the center of a 3D kernel composed of human body geometry. The training dataset consists of density maps obtained from CT images and the reference voxelwise S-values generated using Monte Carlo simulations. Accordingly, specific S-value kernels are inferred from the trained model and whole-body dose maps constructed in a manner analogous to the voxel-based MIRD formalism, i.e., convolving specific voxel S-values with the activity map. The dose map predicted using the DNN was compared with the reference generated using MC simulations and two MIRD-based methods, including Single and Multiple S-Values (SSV and MSV) and Olinda/EXM software package.

**Results:**

The predicted specific voxel S-value kernels exhibited good agreement with the MC-based kernels serving as reference with a mean relative absolute error (MRAE) of 4.5 ± 1.8 (%). Bland and Altman analysis showed the lowest dose bias (2.6%) and smallest variance (CI: − 6.6, + 1.3) for DNN. The MRAE of estimated absorbed dose between DNN, MSV, and SSV with respect to the MC simulation reference were 2.6%, 3%, and 49%, respectively. In organ-level dosimetry, the MRAE between the proposed method and MSV, SSV, and Olinda/EXM were 5.1%, 21.8%, and 23.5%, respectively.

**Conclusion:**

The proposed DNN-based WB internal dosimetry exhibited comparable performance to the direct Monte Carlo approach while overcoming the limitations of conventional dosimetry techniques in nuclear medicine.

## Introduction

Personalized medicine is a new paradigm aiming at improving healthcare while lowering the costs, thus offering great potential for patient-specific diagnosis and optimal treatment [1]. Precision medicine aims at shifting from the current one-size fits-all strategy to an individualized model. Dose calculation in nuclear medicine is tightly linked to this approach [2]. In this framework, personalized dose estimation is crucial for optimizing clinical procedures while minimizing the risk of radiation-induced toxicity [3].

In current clinical practice, patient dose monitoring is commonly based on simplified models, such as those derived by the Medical Internal Radiation Dose Committee (MIRD) formalism [4]. The traditional MIRD technique is based on organ-level dosimetry using time-integrated activity and radionuclide S-values, which represents the mean absorbed dose to a target organ per radioactive decay in a source organ. These quantitative parameters are modeled based on a reference computational model. This approach assumes a uniform activity distribution within each organ and ignores individual anatomical characteristics. To cope with inter-subject variability of anatomical features, the organ-level dosimetry approach was later extended by developing habitus-specific and patient-specific computational models [5–9]. Furthermore, voxel-based dosimetry techniques have been developed, including dose point kernel [10] and voxel S-value (VSV) [4] approaches. Unlike probabilistic methods, dose point kernel is a deterministic approach that calculates the radial absorbed dose distribution around an isotropic point source in a homogeneous water medium [11, 12]. Voxel-level MIRD schema is defined as a 3D voxel matrix representing the mean absorbed dose to a target voxel per unit activity in a source voxel embedded in an infinite homogeneous medium using Monte Carlo (MC) simulations. However, voxel-based dose calculation should in principle take into account non-uniform activity distribution of the radiotracer, the heterogeneity of the medium consisting of different material compositions, e.g., lung, soft tissue, and bone, is ignored. In this regard, direct MC simulations, deemed the gold standard for implementation of a reliable dose calculation framework in clinical setting, enable accurate estimation of whole-body dose map [13, 14]. Though MC simulation takes into account the non-uniform activity distribution and heterogeneity of patient-specific anatomical features, it suffers from expensive computational burden. A number of previous works reported on the use of MC simulations in the context of personalized dosimetry in nuclear medicine [15–17]. Hybrid PET/CT or SPECT/CT images are fed into the MC simulator to model energy deposition of radiation emitted from the injected radiotracer considering the patient-specific anatomy and voxelwise activity distribution obtained from CT and PET/SPECT images, respectively. Several works focused on reaching an optimal compromise between accurate voxel-scale dosimetry and the computational burden [18, 19]. Khazaee Moghadam et al. proposed a tissue-specific dose point kernel approach implemented on a stylized phantom [20]. Lee et al. extended further this idea by applying this methodology on real patient data [21]. They considered multiple material densities for internal dose calculation by providing multiple voxelwise S-value kernels for various media with different densities according to human body tissues. This enabled to provide multiple voxel-scale dose maps in an analogous manner to the MIRD calculations. Consequently, each density-specific dose map was multiplied by the corresponding binary mask of the given density regions obtained from CT-based segmentation, thus enabling the calculation of the final dose map by superposition of the multiple density-specific dose maps. This method improves the accuracy of dosimetry calculations compared with the single voxel S-value approach, but relies on a basic assumption that energy depositions in each voxel arise mainly from self-absorption. This simplification introduces an extra error on the estimated dose distribution, particularly in the boundary of tissues with different densities.

Accurate patient-specific dosimetry is becoming a must taking advantage of advances in targeted radionuclide therapy and theranostic imaging [2]. In personalized dosimetry, MC simulation is still considered the most accurate technique and the de facto reference standard for research application. Yet, this approach is not employed in routine clinical procedures owing to the heavy computational burden. Deep learning emerged as a promising technique in the area of computer vision and image processing, exhibiting superior performance over conventional state-of-the-art methods in medical image analysis in PET and SPECT imaging, including attenuation and scatter correction [22–24], low-count image reconstruction [25–27], and automated image segmentation [9, 28]. More recently, deep learning approaches were employed for radiation dose estimation. Mardani et al. introduced a dose distribution prediction method in external beam radiation therapy using a multi-layer convolutional auto-encoder architecture [29]. Nguyen et al. used a U-Net architecture for clinical treatment plan optimization to improve the treatment plan quality and uniformity while reducing the computational time [30]. Ma et al. implemented a deep learning method to provide isodose features for modulated arc therapy treatment plans [31]. Kearney et al. proposed a 3D fully convolutional dose prediction algorithm for prostate stereotactic body radiotherapy patients [32].

For effective training of a deep learning algorithm, well-defined ground truth is an essential ingredient [33]. In the abovementioned seminal works, the ground truth was obtained from a substitute of MC dosimetry for the training of the networks that may bear some inaccuracies owing to the simplifications in physical models [34]. To address this limitation, Lee et al. used a U-Net deep neural architecture for internal dosimetry where the training ground truths were obtained from direct MC simulation [35]. They fed CT images, representing patient structural features, and static PET images, representing the activity distribution, into the network as input to predict a 3D dose map rate. Gotz et al. set out to estimate dose maps of patients who received ^177^Lu-PSMA using a modified U-Net network [36]. In this work, the training datasets consisted of a two-channel input, including CT images (i.e., patient-specific density map), MIRD-based voxel-scale dose map obtained from SPECT images, and the ground truth obtained from direct MC simulations. In these two works, the deep learning networks were trained using whole-body dose maps obtained from direct MC simulations. However, generation of a comprehensive training dataset in this manner would be challenging owing to the prohibitive computational burden of MC calculations. Hence, these works either relied on a limited number of training samples or made some approximations that could affect the accuracy of the proposed models. Lee et al. reported that the time required for a single full whole-body MC simulation exceeds 4704.03 h using a CPU with four cores and 16 GB RAM [35]. However, GPU-based MC simulations have been recently proposed to overcome this challenge [37–39]. In this regard, we proposed a novel methodology to estimate whole-body dose distributions using a deep convolutional neural network, wherein unlike previous studies, generation of training datasets is no longer a bottleneck. The proposed dose map generation framework consists of two steps. In the first step, a deep neural network (DNN) is employed to predict dose distribution kernels, wherein the training dataset consists of only density maps obtained from CT images as input and the corresponding dose distribution kernel for a point source with unit activity obtained from MC simulations as output. In this approach, the simulation time for generating a ground truth (dose distribution map around the central voxel source) covering the annihilation photon mean free path is about 8000 times less than that required for whole-body MC simulations. This strategy makes it possible to provide a diverse and extensive training dataset. In addition, this approach would reduce the complexity of the training process as the DNN model should learn simpler features corresponding to a point source distribution compared with direct translation from hybrid density/activity maps to absorbed dose map. In the second step, specific dose distribution kernels predicted by the trained model are convolved with the activity map (here time-integrated activity from dynamic PET images) to generate the final whole-body dose map, in a manner analogous to the voxel-based MIRD formalism.

## Materials and methods

### Method description

**Fig. 1:**
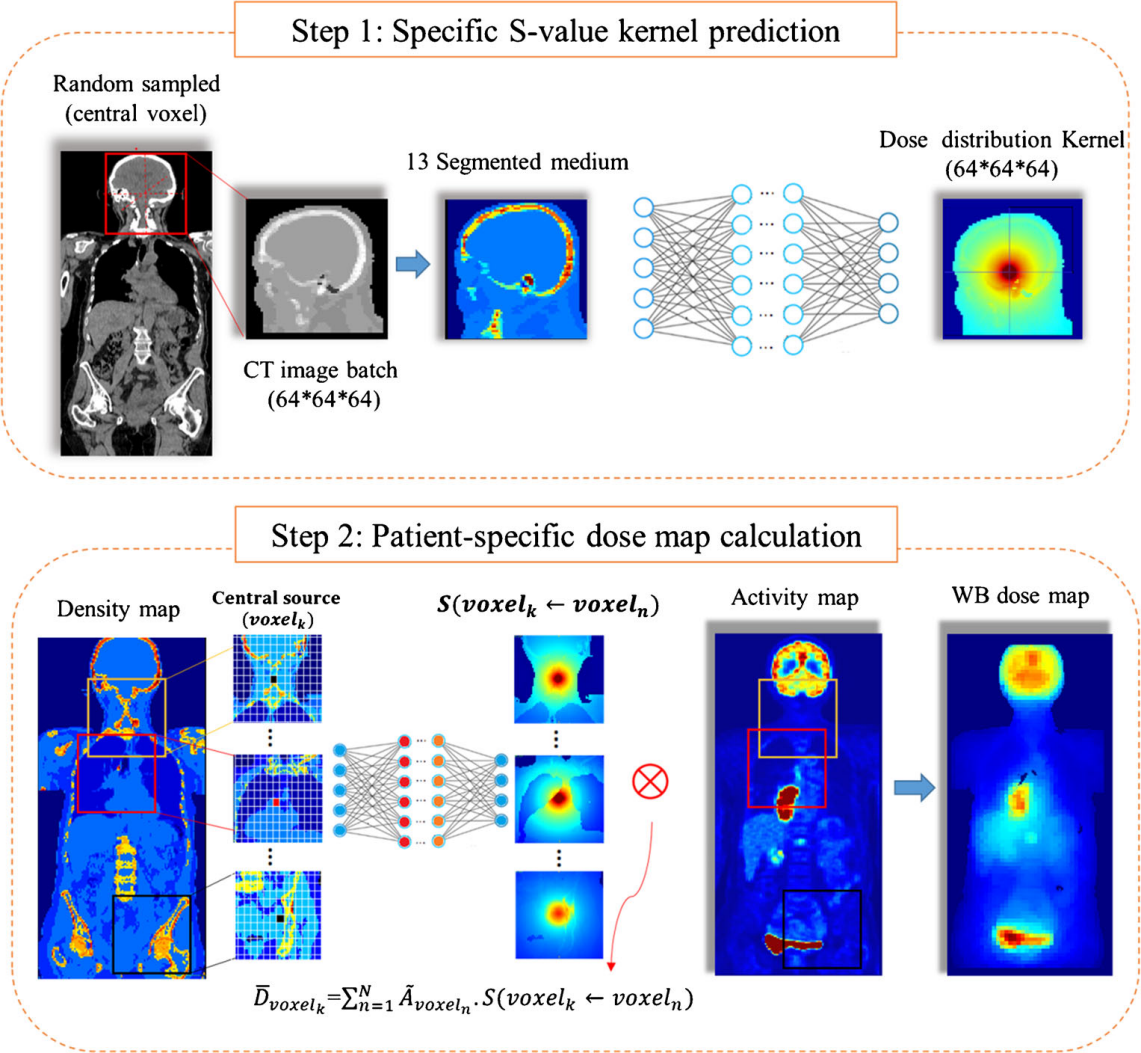
Schematic representation of the voxel-scale dosimetry procedure. The top and bottom panels show the deep learning-based specific S-value kernel prediction and MIRD-based voxel dosimetry formalism

Direct MC simulations, wherein the 3D hybrid PET/CT or SPECT/CT images are fed into a simulator to produce the whole-body dose distribution, are regarded as the gold standard approach. The computational burden of direct MC simulations for building a comprehensive and large training dataset is prohibitive. Hence, we split the direct process into two main parts as schematically illustrated in Fig. 1. The idea is inspired from the MIRD-based voxel-scale dosimetry formalism [4] where a single voxel S-value kernel is convolved with the activity map (e.g., PET images) to produce a whole-body dose map (Eq. 1). In the present study, we extended this idea through estimation of the specific kernels according to the density map obtained from patient’s CT images. Analogous to the MIRD-based voxel-scale dose kernel, we generate specific kernels, i.e., *S*(voxel_k_ ← voxel_h_) in Eq. 1, in such a way that the central voxel contains the unit activity of given a radiotracer, where the surrounding medium is defined based on the patient density map.

The principle of the reciprocity theorem states the reversibility or bilateralism of the interactions upon location interchange of the source and target in a uniform isotropic model. Loevinger introduced this theorem to dose calculation problems in a uniform homogenous medium [40]. Cristy reported that the reciprocity theory is warranted in heterogeneous computational phantoms for photons [41]. We extended this theory to heterogeneous media by applying a source to target correction factor of the energy-absorption coefficient ratio [42]. Since the deposited energy in each voxel depends on the energy fluence multiplied by the mass energy-absorption coefficient of the medium [43], we modified the conditions of location interchange of source and target tacking into account the ratio of the energy-absorption coefficient of target voxel to the source voxel. The reciprocal energy fluence is assumed to be approximately equal for annihilation photons with dominant Compton scattering interaction.

To generate the specific kernels, the distribution of deposited energy around the source voxel was calculated using MC simulations. The size of the kernel depends on the type of radiotracer, i.e., decay mode and energy spectrum. In this work, we defined the size of kernels as 19.2 × 19.2 × 19.2 cm^3^ where the mean free path of annihilation photons in human tissue has been reported to be about 7 cm [44].

1 $$ \overline{D}\left({\mathrm{voxel}}_{\mathrm{k}}\right)=\sum \limits_{h=0}^N{\overset{\sim }{A}}_{{\mathrm{voxel}}_{\mathrm{h}}}.S\left({\mathrm{voxel}}_{\mathrm{k}}\leftarrow {\mathrm{voxel}}_{\mathrm{h}}\right) $$

In the first step, we employed a DNN to predict the specific energy deposition kernel when the source voxel is located in the center of the kernel (Fig. 1). The input data for the training is 3D volume density maps while the corresponding output is 3D volume dose map obtained from MC simulations. To prepare the input dataset for training, single voxels were randomly sampled from whole-body CT images and the surrounding volumes (19.2 × 19.2 × 19.2 cm^3^) were extracted into 64 × 64 × 64 matrices to generate the input samples. Given the input matrices, the MC simulator was employed to produce the dose distribution kernel considering a unit activity at the center of each matrix. In other words, the training of the model was performed for single-point sources located in various positions within the density volume map, i.e., whole-body CT images. Hence, to produce a comprehensive training dataset covering different anatomical sites, we randomly sampled voxels from different whole-body CT images and the surrounding volumes were extracted to generate the input samples. In the second step, the whole-body dose map was calculated by voxelwise convolution of the specific kernels with the activity map (Eq. 1). Hence, we inferred the specific dose distribution kernel for each source voxel, i.e., *S*(voxel_k_ ← voxel_h_), using our trained neural network model. We estimated the whole-body dose map in an analogous way to the MIRD voxel formalism, which convolves a single S-value kernel with each voxel in the activity map, yet using specific S-values kernel for each voxel.

### Deep neural network architecture

**Fig. 2:**
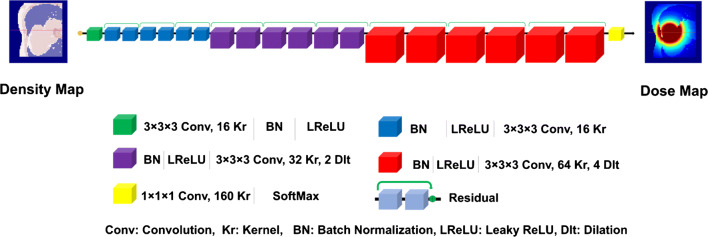
Schematic diagram of the ResNET architecture

In this work, the ResNET [45] architecture implemented on TensorFlow platform, composed of 20 convolutional layers with dilation convolution operations within different levels of feature extraction, was utilized. The dilation factor supports the expansion of the receptive field-of-view without resolution loss by increasing the space between original kernel elements. For low-level feature extraction, a dilation factor of zero was used within the first seven layers, a dilation factor of two within the second seven layers for medium-level feature extraction, and a dilation factor of four within the last six layers for high-level feature extraction. Leaky rectified linear unit (LReLU) was used as activation function. The ResNET architecture benefits from 9 residual blocks, which results in a large number of receptive fields and improves the process of feature extraction and network convergence (Fig. 2).

For the training of the model, pairs of CT density images and deposited energy kernels were considered input/target, respectively. The ResNET model with a 3D spatial window equal to 3 × 3 × 3 voxels was used. The following setting was used for the training of the network: learning rate = 0.001, sample per volume = 1, optimizer = Adam, and decay = 0.0001. The optimization of the network was carried out based on the L2 loss function.

### Data preparation

To prepare the training dataset, density maps were extracted from CT images. CT Hounsfield units (HUs) have a strong correlation with electron density, and consequently with the mass density of the medium. We converted HU values to mass density using the methodology proposed by Schneider et al. which established linear multi-regression models for the segmentation of CT images into different tissue densities [46]. We extracted density maps consisting of 13 tissue densities, including air, lung, fat, soft tissue, and bone where values higher than 100 HUs were divided into eight discrete density values. Afterward, the whole-body density maps were resampled to 3 mm voxel size in three dimensions. To build the ground truth data, MC simulations served as standard of reference. The MCNP transport code [47] was employed for the generation of energy deposition kernels, i.e., specific voxel S-values. To this end, one voxel was randomly sampled from the whole-body density maps and a 3D matrix of 64 × 64 × 64 voxels around the central voxel was extracted. This matrix, representing a heterogeneous medium of patients’ anatomical structures, was directly imported to the MCNP code. The material compositions of 13 segmented tissues were defined based on Schneider et al. [46]. The central voxels of the extracted 3D matrix were defined as source location with uniform distribution of fluorodeoxyglucose (^18^F-FDG). Since the resolution of the activity distribution (here PET images with an average resolution of 3 mm) determines the spatial accuracy of dosimetry estimations, we adopted the same resolution for the calculation of dose maps. The energy spectrum of emitted positrons was taken from [48], where the positron energy spectrum follows a Fermi distribution with an average of 242.8 KeV and maximum energy of 633.5 KeV. The output of MC simulations consists of 3D kernels (64 × 64 × 64) with 3 mm resolution using energy deposition mesh tally in unit of MeV/cm^3^ per particle. Three million particles were tracked to reach a statistical uncertainty less than 4% in the border voxels at about 10 cm away from the central voxel.

### Clinical studies

To provide whole-body dose maps from an activity map based on Eq. 1, a specific S-value kernel is required for each single voxel of the activity map. Whole-body unenhanced CT images of 24 patients acquired on Siemens Definition Edge system were used for the training of the model (generation of the training dataset). The study protocol was approved by Geneva Ethics Committee and all patients provided written informed consent. For evaluation of the model, hybrid PET/CT image sets consisting of a low-dose CT scan and dynamic whole-body PET scans were employed. The hybrid PET/CT image sets were acquired on a Siemens Biograph mCT scanner using a dynamic scanning protocol at 13-time points after intravenous injection of ^18^F-FDG [49, 50]. PET scanning was conducted using continuous bed motion scan at ever increasing time intervals. PET image reconstruction was performed using 3D iterative ordinary Poisson OSEM (3D-OP-OSEM) algorithm with a voxel size of 4.07 × 4.07 × 3 mm.

### Dose map calculation

To estimate whole-body voxelwise absorbed dose, the trained model was fed with patient-specific density maps to generate the specific dose distribution kernels, *S*(voxel_k_ ← voxel_h_), for each single voxel (i.e., voxel_k_) in the PET image, wherein the corresponding voxel in CT images and its surrounding 64 × 64 × 64 voxels were considered the input density map. The predicted specific S-values were corrected by element-wise multiplication of the ratio of the energy-absorption coefficient of the target voxel to the source voxel obtained from [51]. Lastly, specific S-values underwent voxelwise convolution with the cumulated activity map to create the whole-body dose map (Eq. 1). The cumulated activity map was calculated by analytical integration of voxelwise time activity curves over 13-time points dynamic PET frames (Eq. 2).

2 $$ {\overset{\sim }{A}}_{\mathrm{T}\mathrm{otal}}=\sum \limits_{i=0}^{13}\left({A}_i+{A}_{i+1}\right).\Delta  {t}_i+\underset{\mathrm{T}}{\overset{\infty }{\int }}{A}_{\mathrm{f}}{e}^{-\lambda t} dt $$

In Eq. 2, ${\overset{\sim }{A}}_{\mathrm{Total}}$ is the total number of disintegrations, *A*_*i*_ is the activity concentration in the source organ obtained from static images at the *i*th time frame, *A*_f_ is the activity concentration in the last time point of measurement, and *λ* is the decay factor of the radionuclide. Bladder voiding schedules were not taken into account. To conduct patient-specific whole-body voxelwise dose estimation, the results were converted in Gy after multiplication by a correction factor of 0.9673 corresponding to the fraction of positron emission for ^18^F.

To evaluate the proposed method, the predicted absorbed dose from the current model was compared against direct MC dose estimation serving as standard of reference and different MIRD-based approaches, including the OLINDA/EXM software (organ-scale MIRD formalism) [52], single voxel S-value (SSV), and multiple voxel S-value (MSV). For organ-level dosimetry, regions-of-interest were manually drawn on CT images to delineate eight organs, namely the brain, heart, kidneys, liver, lungs, spleen, bone, and bladder. Lesions identified on PET images were segmented using a fixed threshold of 42% of SUV_max_ and manually edited to remove the background and include necrotic regions. The kinetic data required by the Olinda/EXM software were calculated from the cumulated activity using Eq. 2 and the masses of organs were modified based on organ masks defined from the segmentation of CT images. SSV and MSV voxel-scale dosimetry was designed based on the MIRD formalism (Eq. 1) where the voxel S-value kernels were generated from MCNP code with the same kernel size used in the previous step, i.e., 19.2 cm in 3D with 3 mm resolution. Ten million particles were simulated to build a 64 × 64 × 64 kernel in an infinite homogenous medium considering a unit activity in the central voxel. In the MSV method [21], the S-value kernels of four different media consisting of soft tissue, lung, and two different densities of bone (with different calcium contents) were simulated.

### Quantitative analysis

Voxelwise mean absolute error (MAE), mean relative absolute error (MRAE %), and root mean square error (RMSE) were calculated between reference and predicted dose maps.

3 $$ \mathrm{MAE}=\frac{1}{vxl}\sum \limits_{v=1}^{vxl}\left|{\mathrm{Image}}_{\mathrm{predicted}}(v)-{\mathrm{Image}}_{\mathrm{ref}}(v)\right| $$

4 $$ \mathrm{MRAE}\left(\%\right)=\frac{1}{vxl}\sum \limits_{v=1}^{vxl}\left|\frac{{\left({\mathrm{Image}}_{\mathrm{predicted}}\right)}_v-{\left({\mathrm{Image}}_{\mathrm{ref}}\right)}_v}{{\left({\mathrm{Image}}_{\mathrm{ref}}\right)}_v}\right|\times 100\% $$

5 $$ \mathrm{RMSE}=\sqrt{\frac{1}{vxl}\sum \limits_{v=1}^{vxl}{\left({\mathrm{Image}}_{\mathrm{predicted}}(i)-{\mathrm{Image}}_{\mathrm{ref}}(i)\right)}^2} $$

where Image_predicted_ stands for the dose map generated by the DNN and Image_ref_ stands for the reference dose map. *vxl* and *v* denote the total number of voxels and voxel index, respectively.

## Results

### Network validation

**Fig. 3:**
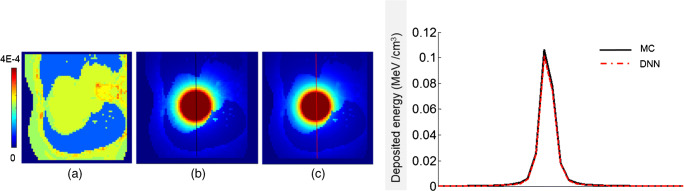
**a** CT-based density map. **b** Reference kernel obtained from MC simulations. **c** Predicted kernel by the DNN model. Line profiles across the S-value kernels (right panel) comparing kernels obtained from MC simulations of DNN model predictions

**Fig. 4:**
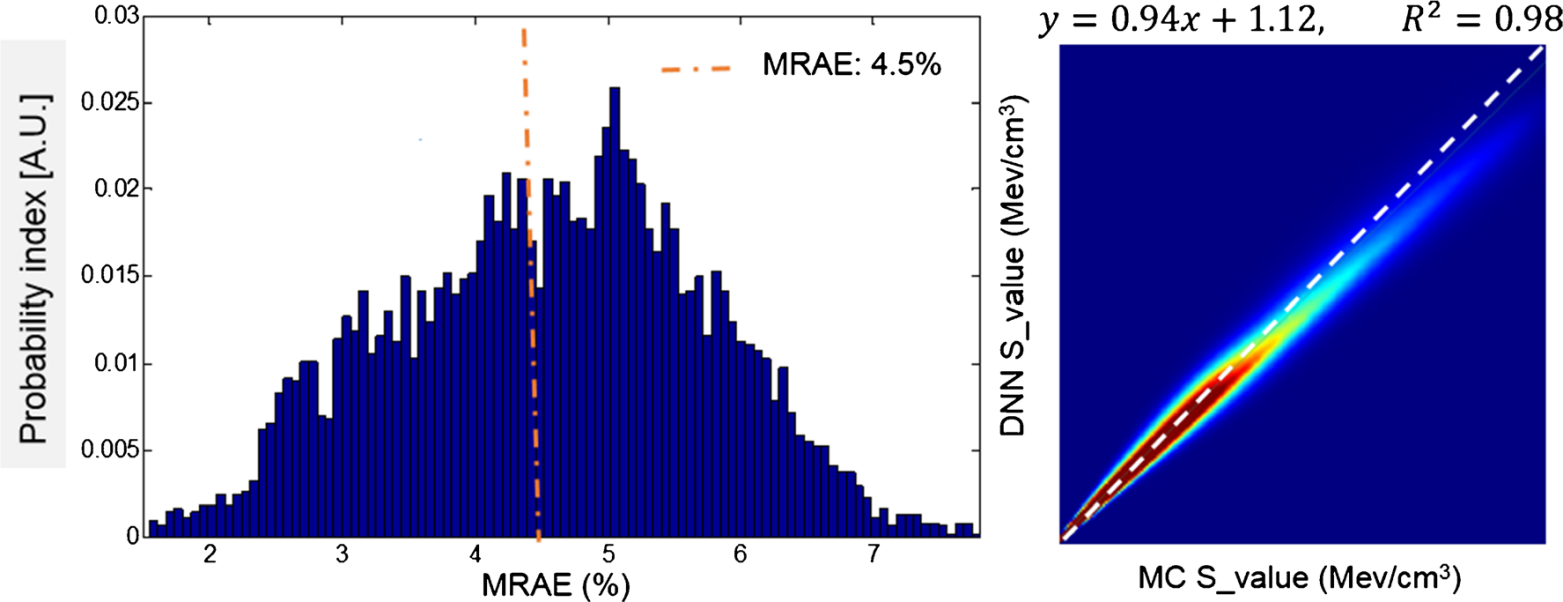
Probability distribution of relative absolute error (RAE) for predicted voxelwise S-value kernels (64 × 64 × 64) with respect to MC simulations (left). A.U. = arbitrary units. Voxelwise joint histogram plot depicting the correlation of predicted kernels with respect to MC simulations (right)

The total number of training dataset consisted of 12′100 pairs of volumetric images of density maps and energy deposition kernels extracted from 24 different CT image sets. The specific voxelwise S-value kernels, obtained from the DNN, were in good agreement with the reference MC kernels. The axial profiles plotted over reference and predicted voxelwise S-value kernels in the lung region are shown in Fig. 3. The mean relative voxelwise difference between the two profiles is about 3.3%. Figure 4 illustrates the comparison of predicted voxel S-values (64 × 64 × 64) against MC simulations for the test case in the lung region with MRAE, RMSE, and MAE of 4.5 ± 1.8 (%), (1.8 ± 0.53) × 10^−5^ (MeV/cm^3^), and (1.8 ± 0.71) × 10^−6^ (MeV/cm^3^), respectively. Furthermore, the voxelwise joint histogram plot depicting the correlation between the predicted kernels and MC simulations is presented, where a coefficient of determination (*R*^2^) of 0.98 was achieved.

### Analysis of dose distributions

**Fig. 5:**
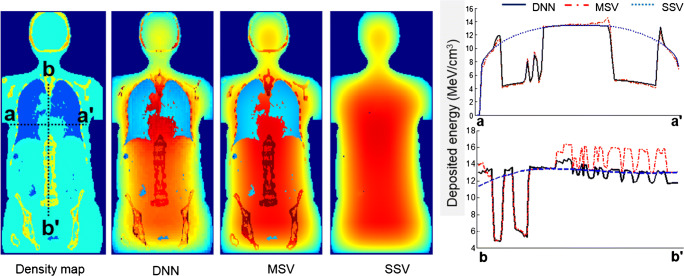
Voxelwise deposited energy (MeV/cm^3^) in a patient-specific computational phantom with unit activity distribution estimated by DNN, MSV, and SSV

To assess the impact of medium heterogeneity on dosimetry results, a whole-body map of deposited energy was generated for a patient-specific computational phantom with unit activity distribution using three different methods, including DNN, MSV, and SSV. In this regard, calculation of patient-specific absorbed dose map (step 2 in Fig. 1) involves filling the patient’s body contour with a unit activity distribution instead of a time-integrated activity map obtained from a dynamic PET series. Dose profiles over axial and coronal slices are illustrated in Fig. 5. It is expected that SSV in medium with densities lower than water overestimates the deposited energy while underestimating the deposited energy for higher density media. The deposited energies obtained from MSV confirm the limitation of this method in the heterogeneous boundaries in the spine area with an average density of about 1.12 g/cm^3^ (b-b’ line profile).

**Fig. 6:**
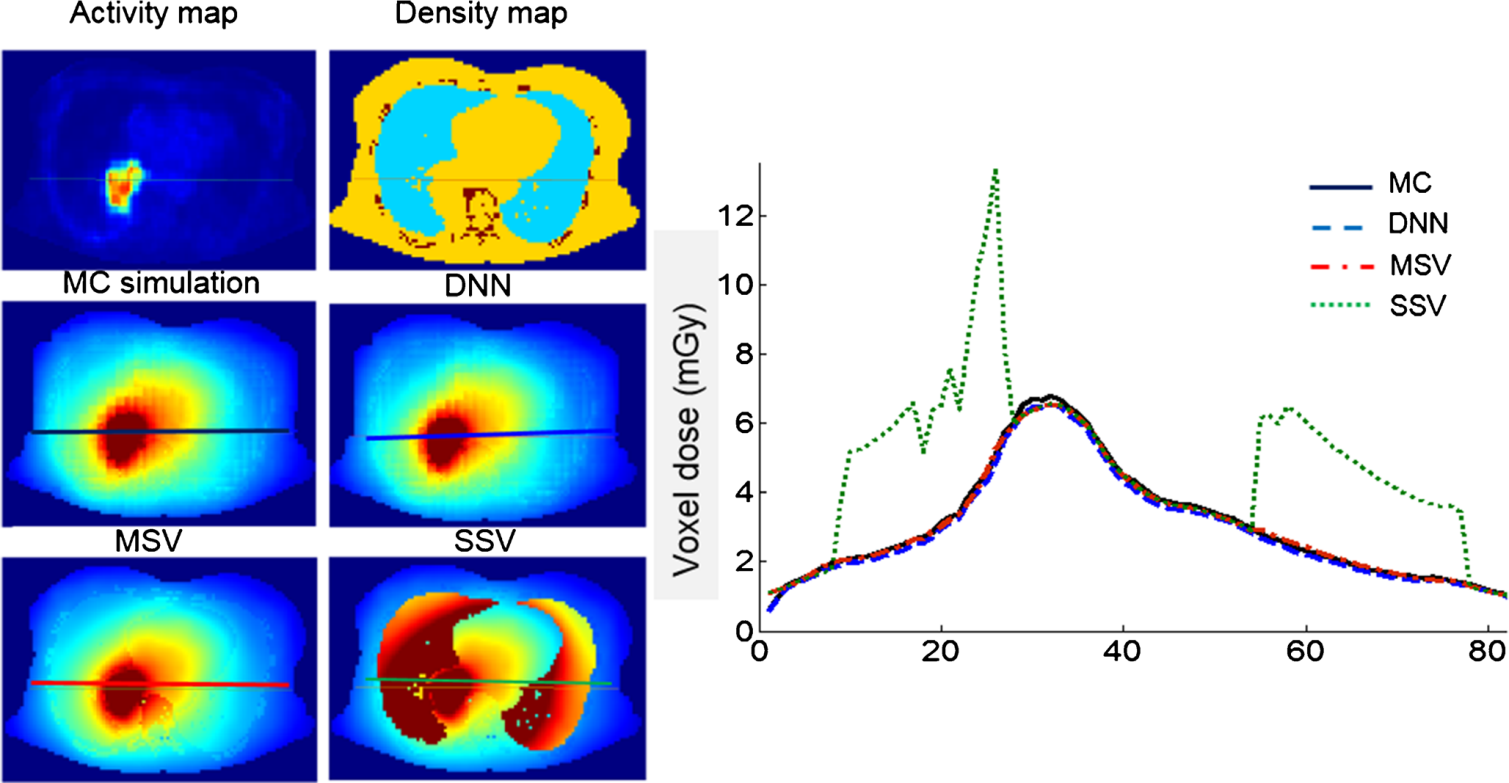
Dose distributions and profiles (right) drawn on axial views comparing dose maps estimated using DNN, MSV, and SSV methods against MC simulations

**Fig. 7:**
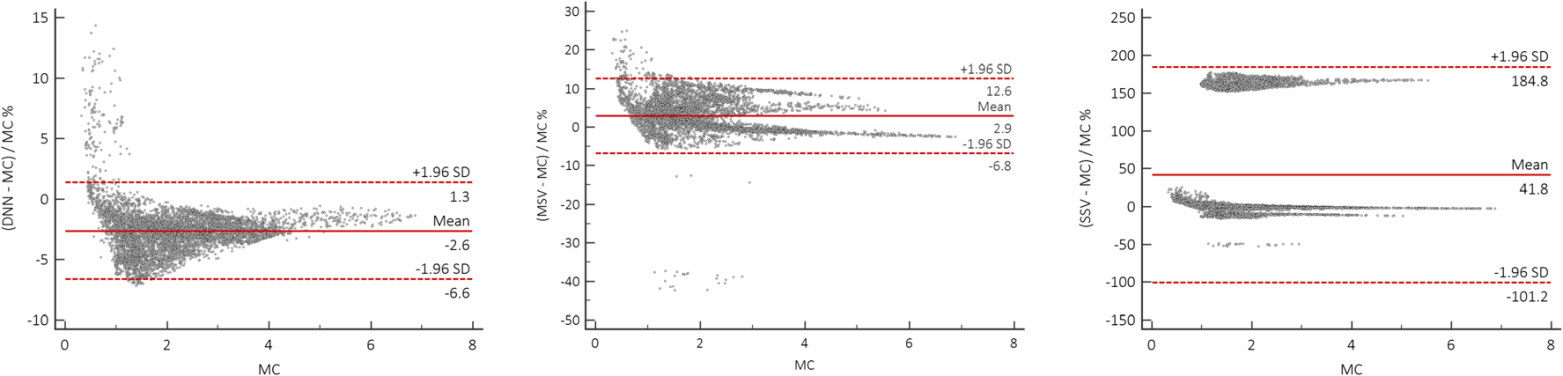
Bland and Altman plots of voxelwise dose differences in the lung region calculated using DNN (left), MSV (middle), and SSV (right) with respect to MC-based calculations serving as standard of reference. The solid and dashed lines denote the mean and 95% CI of the dose value differences, respectively

**Fig. 8:**
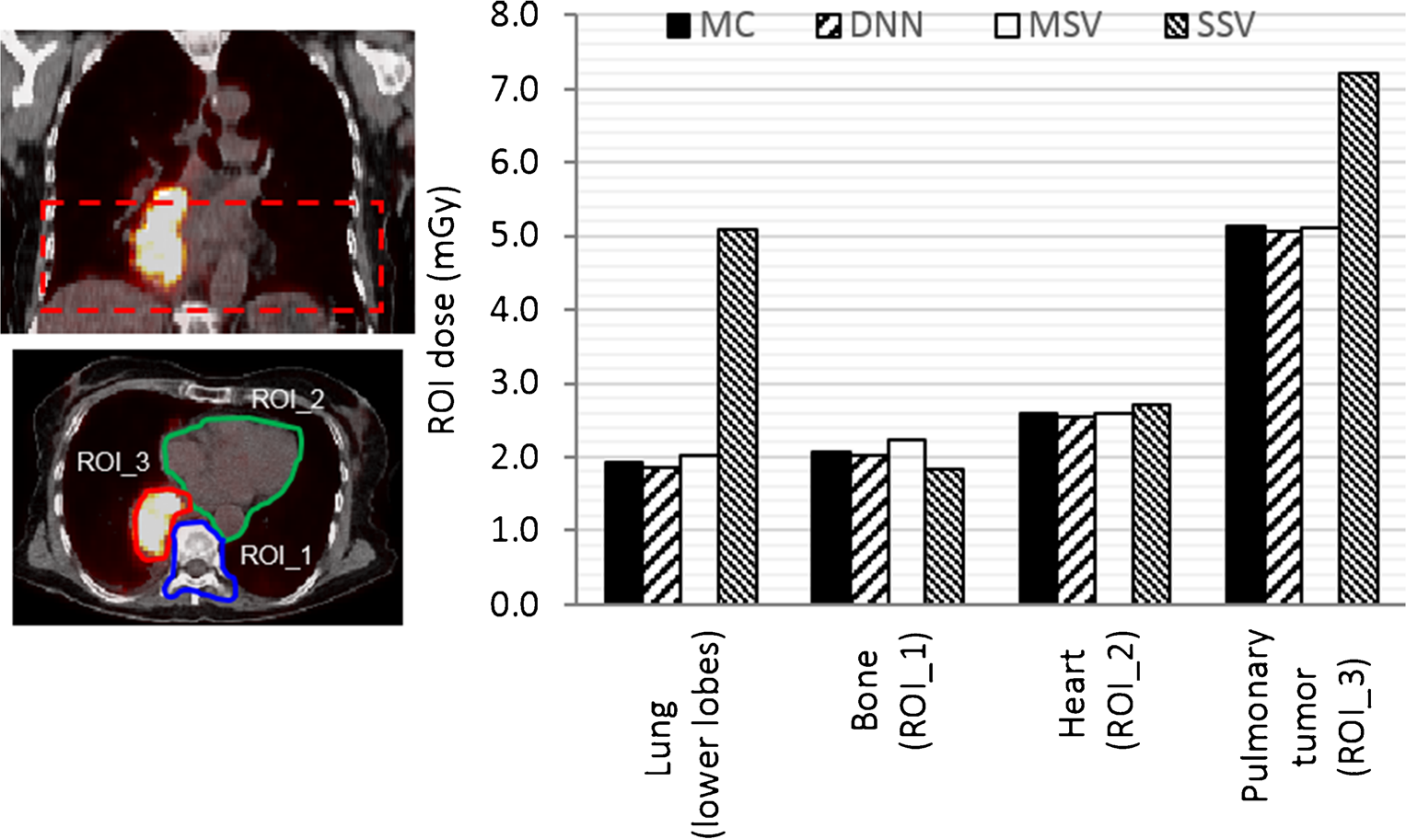
Anatomical region for dose evaluation (top left), axial view of delineated VOIs (bottom left). Average absorbed doses in defined VOIs obtained using DNN, MSV, and SSV compared with MC calculations (right)

The voxelwise dose maps predicted by DNN and estimated using MIRD-based methods, including SSV and MSV, were compared with the results obtained from MC simulations for a patient diagnosed with lung adenocarcinoma having a pulmonary tumor of about 120 g. Figure 6 displays a representative dose profile drawn on axial views comparing dose maps estimated from DNN, MSV, and SSV against MC simulations. To quantify the agreement between the different methods with respect to the standard of reference, Bland-Altman plots compare absorbed doses calculated using DNN, MSV, and SSV with MC-based calculations. Figure 7 illustrates the bias and variance with 95% confidence interval (CI) of these methods against the standard of reference method, where the data points reflect the percent difference of voxelwise dose values. The results show that the lowest absorbed dose bias (2.6%) and the smallest variance (CI: − 6.6%, + 1.3%) were achieved by the DNN approach. In addition, the results obtained using MSV demonstrated good agreement with the ground truth (absorbed dose bias of 2.9% and variance of CI: − 6.8%, + 12.6%), except in some regions corresponding to heterogeneous boundaries. Conversely, SSV showed significant discrepancy compared with the reference in the lung and bone regions. In the lung region illustrated in Fig. 8 (top left), four VOIs over the heart, bone, lower lobes of the lungs, and pulmonary tumor were drawn on fused PET/CT images to perform quantitative analysis of absorbed doses within the VOIs. The mean absolute relative errors of estimated absorbed doses between DNN, MSV, and SSV against MC simulations were 2.6 ± 0.94%, 3 ± 3.5%, and 49 ± 68%, respectively.

**Fig. 9:**
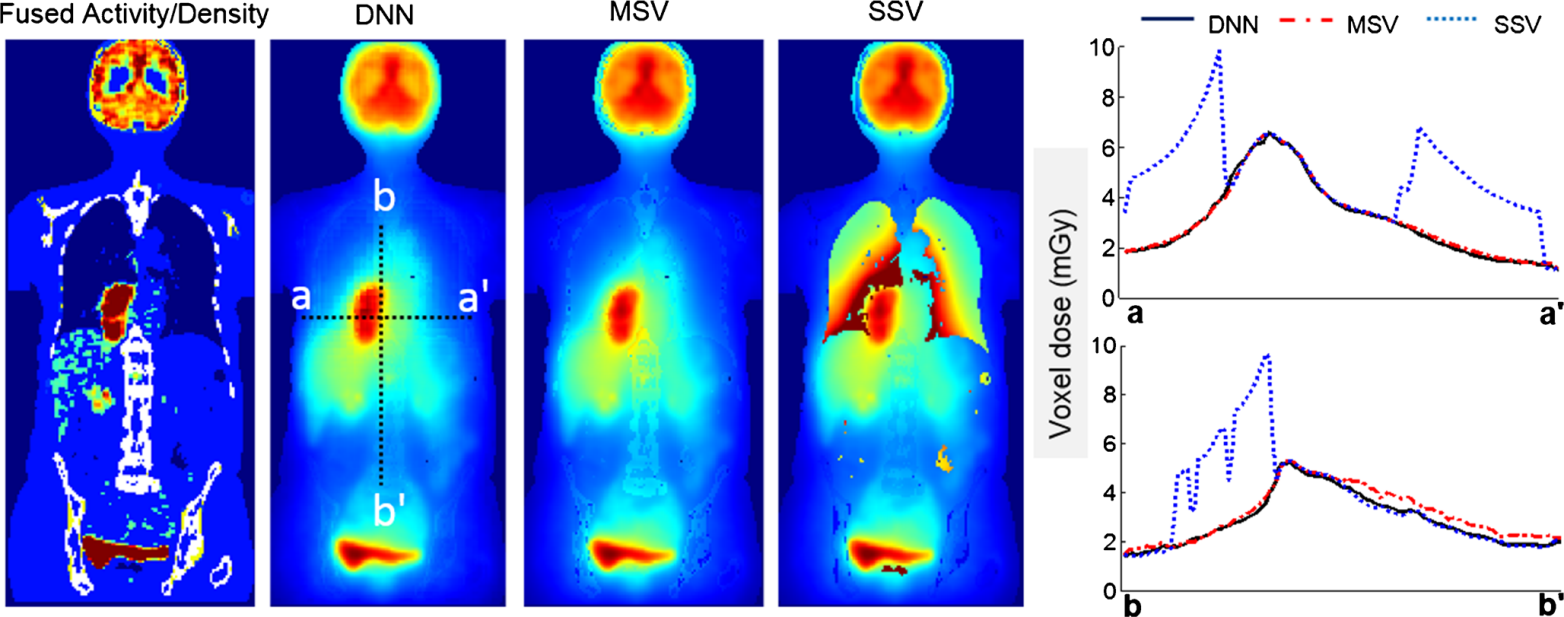
Voxelwise dose maps estimated using DNN, MSV, and SSV along with horizontal and vertical profiles drawn on the coronal view

**Fig. 10:**
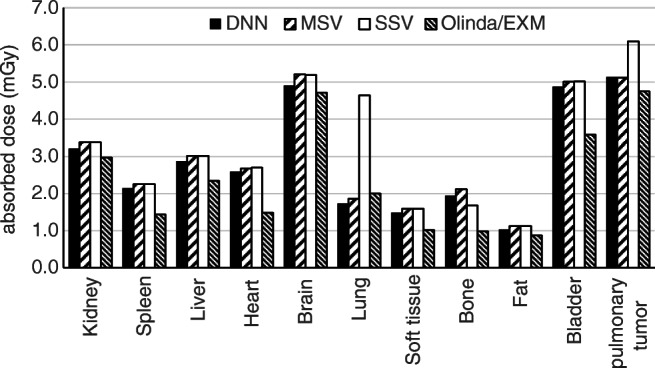
Whole-body organ-level absorbed doses estimated using DNN, MSV, SSV, and Olinda/EXM software

Whole-body voxelwise absorbed dose estimations based on time-integrated activity and patient-specific anatomy obtained from a dynamic PET/CT scan are presented in Fig. 9 along with two profiles plotted over axial and coronal views. Organ-level dosimetry was extracted from the dose maps obtained from DNN, MSV, and SSV methods and compared against a commercial organ-based MIRD dosimetry software, i.e., Olinda/EXM (Fig. 10). In most organs, Olinda/EXM underestimates the absorbed dose compared with other voxel-based methods except for lung. The MRAE between organ doses estimated by DNN method and MSV, SSV, and Olinda/EXM were 5.1%, 21.8%, and 23.5%, respectively.

## Discussion

Despite the paramount importance of personalization in routine clinical setting, this paradigm is still in its infancy, and in the literature, only a few studies have addressed this issue. In this work, we propose a novel methodology to perform personalized radiation dose quantification, which is applicable in various nuclear medicine procedures including diagnostic, therapeutic, and theranostics. The current methodology has been employed in PET imaging dosimetry using ^18^F-FDG radiopharmaceutical, as the proof of concept. We developed a MC-based whole-body voxel-level dosimetry approach to enable studies that might provide answers to controversies on whether voxel-based dosimetry is superior to the mean absorbed dose approach [53]. Deep learning algorithms have been deployed to solve complex real-life problems by translating the fundamental physics behind the problem into the computer vision domain. In this work, we extended the core idea of the voxel-level MIRD dosimetry formalism by using DNN algorithms to predict medium-specific S-value kernels instead of using a single kernel obtained from deposited energy in a homogenous soft tissue medium. The size of the kernel was 19.2 cm where the distance from the central voxel to the border is more than the mean free path of annihilation photons (511 KeV). In a kernel of 19.2 cm in three dimensions representing voxelwise deposited energy in an infinite soft tissue medium obtained from MC simulations, the ratio of the deposited energy at the border of the kernel to the central source voxel is about 10–4 order of magnitude confirming adequate size of the kernel. Although increasing the size of the kernel up to three mean free paths of annihilation photons from the center of the kernel can improve the accuracy of dose estimation, it would induce considerably longer simulation time. The resolution of the kernel was defined based on regular axial resolution of PET images. The statistical uncertainty of MC simulations was less than 4%. It is obvious from Fig. 2 that even in the border of the S-value kernels, the noise level owing to statistical uncertainty is not significant. To benchmark our assumption for extending the reciprocal theory to heterogeneous medium, we simulated a simple geometry consisting of soft tissue, bone, and lung materials and calculated the deposited energy when the source and target were locally interchanged. The deposited energies calculated using the reciprocal theory were within 5% of those calculated by simulations.

The predicted 3D kernels exhibited good agreement with MC simulations with a MRAE of 4.5%. The DNN predicted S-value kernel underestimates the ground truth as illustrated in the joint histogram analysis. The comparison of the summation of the predicted 3D kernel against the summation of MC S-value kernel, as an index of total energy deposition in the medium, showed an overall 4% underestimation. Since the deposited energy follows an inverse square law with respect to the distance from the source, S-value kernels bear a very broad dynamic range of intensities. Hence, we implemented a nonlinear intensity normalization using a sigmoid function before feeding the kernels into the network. Owing to the nonlinear behavior of the sigmoid function, increased prediction errors were observed for certain intensity values after applying inverse sigmoid function. However, the model performed overall much better using nonlinear normalization.

Voxelwise dose comparison between the proposed approach and conventional techniques revealed the limitations of SSV and MSV for internal dosimetry calculations. Since media with higher densities inherently contain more photon interactions, this causes higher energy deposition in the voxels, and the summation on S-value kernels of higher density media has higher values compared with those with lower densities. The profiles of absorbed dose showed that SSV overestimated the deposited energy within media with a density lower than soft tissue (e.g., lung) (Fig. 5). This concept applies to densities higher than soft tissue, such as bone where SSV underestimated the deposited energy. None of the abovementioned seminal works compared their results with the MSV approach which showed a good agreement with direct MC simulations. However, since this approach relies on the assumption that most absorbed doses are contributed by self-absorption, dose estimation errors are commonly observed at the boundaries of heterogeneous media (see Fig. 7 where few data points significantly deviated from the reference), which is not clinically important. Furthermore, MSV underestimated or overestimated the absorbed dose in VOIs with small size depending on the medium density. Figures 5, 8, and 9 confirm that MSV overestimated the results with respect to the ground truth in bones. These errors were predictable since the total deposited energy in the soft tissue kernel is 54% higher than that in lung kernel with the same size, while this difference is about − 34% between soft tissue and cortical bone kernels. This limitation causes significant errors in absorbed dose estimation for small size lesions in media with different mass densities, e.g., pulmonary nodules, which is a critical issue in targeted radionuclide therapy. In addition, application of the MSV method is restricted to radiotracers with higher positron energy, since taking only self-absorption into account does not fulfill the requirements of accurate internal dosimetry. The Bland-Altman analysis demonstrated the lower bias and variance of DNN against MSV and SSV. The data points of the DNN method beyond the CI correspond to voxels at the boundary of body contour having no impact on dose calculation results. In addition, the data points of MSV method beyond the CI belong to voxels with heterogeneous boundaries, while for SSV, three separate regions were formed corresponding to three different media. In nuclear medicine practice, knowledge of organ-scale absorbed dose according to the different radiosensitivity of organs is required. Olinda/EXM is a commercial software package enabling estimation of organ-level absorbed doses according to the MIRD formalism. For the studied patient, it was observed that organ-level dosimetry leads to underestimation of absorbed dose compared with voxel-level approaches, except the lungs, as a result of ignoring the non-uniformity of organ activity distribution and inter-subject variability of anatomical characteristics (Fig. 10). Another limitation of this software is the use of isolated sphere model for tumor dosimetry. This latter assumes that tumors are spheres with unit density and uniform activity distribution and there is no information about the cross-dose from a tumor to other organs or from other organs to a tumor. Because of this limitation, in the case study with a pulmonary tumor, we determined the total number of disintegrations within the lung and tumor as input kinetic parameters of the lung in Olinda/EXM, which led to an overestimation of lung self-absorbed dose by Olinda/EXM. Conversely, the underestimation of tumor dose lies in the fact that only self-absorbed dose is considered in Olinda, whereas cross-irradiation is ignored [54, 55]. Absorbed doses in most organs considered soft tissue were almost similar when using MSV and SSV techniques. MSV was able to correct the SSV errors in regions with a density different from soft tissue.

The importance of accurate patient-specific voxel-scale internal dosimetry is rapidly growing thanks to recent advances in targeted radionuclide therapy and theranostics. Considering the advantages of voxel-level dosimetry in molecular radiotherapy in terms of providing dose indices, such as dose volume histograms, we developed a methodology for voxelwise dosimetry. The execution time for building a whole-body voxel dose map is less than 0.1% of the time required for direct MC simulations. However, the computational time is longer than that of MSV because it has one additional component for inferring the specific S-value kernels. The total computation time for the first step is about 0.7 h using NVIDIA GEFORCE RTX 2080 Ti platform, whereas the required time for the convolution process is about 0.1 h on a 10-core CPU and 32 GB RAM. The results presented in this work demonstrated that MSV provides reasonable accuracy for dose estimation in diagnostic nuclear medicine procedures. However, due to its limitations, it introduces significant uncertainties which might limit its adoption in therapeutic applications. The proposed method is robust and accurate and suitable for direct transfer to other molecular imaging modalities. Its advantages compared with other deep learning-based dosimetry techniques reported in the literature [35, 36] are that it does not require whole-body dose maps for the training step. In addition, a single-trained model for a given radionuclide could be employed for all compounds labeled with this radionuclide. Furthermore, the fundamental principles and/or underlying physics of energy deposition have been considered in our model. The latter depends directly on the energy-absorption coefficient of the target voxel and the probability of Compton scattering, which depends on the density of the medium. Previous works did not explicitly incorporate in their model Compton scattering and its contribution to the overall absorbed dose. In this regard, deep learning algorithms were employed to predict the absorbed dose map from the density/activity maps using an end-to-end scheme without explicitly modeling the underlying physical principles (Compton scattering and cross-irradiation). More importantly, we developed a simple network with a single input/output channel featuring detailed modeling of the underlying physical interactions, which enables efficient and versatile training of the algorithm with minimal risk of overfitting. Owing to the simple but efficient deep learning-based core of the proposed framework (smaller number of trainable parameters compared with end-to-end image translation), the model provides an accurate and robust solution using a small training dataset.

This work bears inherently some limitations that should be acknowledged, among them the long time required for simulation-based generation of ground truth dose maps. First, the size of S-value kernel is about one mean free path of annihilation photons. Second, extending the reciprocal theory to heterogeneous media is not straightforward. However, we proved the efficacy of the concept using a simple simulation study. Third, the effect of the limited size of the training and validation dataset warrants further investigation. However, a single patient study was presented as a proof of concept. Unlike organ-level dosimetry that is inherently subject-sensitive, estimation of voxelwise dose distribution based on the voxel-based MIRD formalism is not subject-sensitive since it depends only on physical parameters (S-value kernel, density map, and activity distribution). In this context, the accuracy of the results depends only on how the S-value kernels are determined. Let us consider that SSV performs well in homogenous media, and the accuracy of this method is not related to the type of medium or the activity distribution. The accuracy of this method directly depends on the S-value kernels applied for voxelwise dosimetry. Likewise, the accuracy of the proposed methodology is linked to the accuracy of the specific S-value kernels while it is not dependent on patient-specific anatomy and activity distribution. Hence, in the first step, we evaluated our S-value prediction voxel-by-voxel to assess the accuracy of our approach (Fig. 4). Lastly, we only provided a model for 18F, yet our method is extendable to all types of radionuclides/radiotracers where transfer learning can be exploited to obviate the need for regeneration of large ground truth dataset for training the network. In particular, for positron-emitting radiotracers with different positron energies, the generation of the ground truth should be repeated for a kernel size equal to the range of positrons. Since the deposited energy outside the positron range is contributed by the interactions of annihilation photons, for any pure positron-emitting radiotracer, the central part of S-value kernels should be replaced with the center of simulated S-value kernels for 18F generated in the current study.

## Conclusion

We proposed a unified methodology for patient-specific voxelwise whole-body internal dosimetry using deep learning algorithms. The comparison of the proposed approach with standard of reference MC simulations revealed very good accuracy with a MRAE of 2.6%. Our technique also outperformed conventional voxel-level and organ-level MIRD-based formalisms. Future work will focus on exploiting the current methodology to generate whole-body voxelwise dose maps in few minutes to serve as Monte Carlo-based ground truth datasets. A network with two-channel inputs consisting of density/activity map pairs and one output channel corresponding to voxelwise dose maps obtained from the previous step is then trained to develop a model for straightforward prediction of whole-body dose maps from hybrid images.

## References

[1] Baumann M, Krause M, Overgaard J, Debus J, Bentzen SM, Daartz J (2016). Radiation oncology in the era of precision medicine. Nat Rev Cancer.

[2] Stabin MG, Madsen MT, Zaidi H (2019). Personalized dosimetry is a must for appropriate molecular radiotherapy. Med Phys.

[3] Zaidi H, Xu XG (2007). Computational anthropomorphic models of the human anatomy: the path to realistic Monte Carlo modeling in radiological sciences. Annu Rev Biomed Eng.

[4] Bolch WE, Bouchet LG, Robertson JS, Wessels BW, Siegel JA, Howell RW (1999). MIRD pamphlet No. 17: the dosimetry of nonuniform activity distributions-radionuclide S values at the voxel level. Medical Internal Radiation Dose Committee. J Nucl Med.

[5] Johnson PB, Whalen SR, Wayson M, Juneja B, Lee C, Bolch WE (2009). Hybrid patient-dependent phantoms covering statistical distributions of body morphometry in the US adult and pediatric population. Proc IEEE.

[6] Akhavanallaf A, Xie T, Zaidi H (2019). Development of a library of adult computational phantoms based on anthropometric indexes. IEEE Trans Radiat Plasma Med Sci.

[7] Na YH, Zhang B, Zhang J, Caracappa PF, Xu XG (2010). Deformable adult human phantoms for radiation protection dosimetry: anthropometric data representing size distributions of adult worker populations and software algorithms. Phys Med Biol.

[8] Xie T, Akhavanallaf A, Zaidi H (2019). Construction of patient-specific computational models for organ dose estimation in radiological imaging. Med Phys.

[9] Xie T, Zaidi H (2019). Estimation of the radiation dose in pregnancy: an automated patient-specific model using convolutional neural networks. Eur Radiol.

[10] Berger MJ (1971). Distribution of absorbed dose around point sources of electrons and beta particles in water and other media.

[11] Kolbert KS, Sgouros G, Scott AM, Bronstein JE, Malane RA, Zhang J (1997). Implementation and evaluation of patient-specific three-dimensional internal dosimetry. J Nucl Med.

[12] Giap HB, Macey DJ, Bayouth JE, Boyer AL (1995). Validation of a dose-point kernel convolution technique for internal dosimetry. Phys Med Biol.

[13] Zaidi H (1999). Relevance of accurate Monte Carlo modeling in nuclear medical imaging. Med Phys.

[14] Gardin I, Bouchet LG, Assie K, Caron J, Lisbona A, Ferrer L (2003). Voxeldose: a computer program for 3-D dose calculation in therapeutic nuclear medicine. Cancer Biother Radiopharm.

[15] Papadimitroulas P, Loudos G, Nikiforidis GC, Kagadis GC (2012). A dose point kernel database using GATE Monte Carlo simulation toolkit for nuclear medicine applications: comparison with other Monte Carlo codes. Med Phys.

[16] Besemer AE, Yang YM, Grudzinski JJ, Hall LT, Bednarz BP (2018). Development and validation of RAPID: a patient-specific Monte Carlo three-dimensional internal dosimetry platform. Cancer Biother Radiopharm.

[17] Ljungberg M, Gleisner KS (2018). 3-D image-based dosimetry in radionuclide therapy. IEEE Trans Radiat Plasma Med Sci..

[18] Dieudonne A, Hobbs RF, Lebtahi R, Maurel F, Baechler S, Wahl RL (2013). Study of the impact of tissue density heterogeneities on 3-dimensional abdominal dosimetry: comparison between dose kernel convolution and direct Monte Carlo methods. J Nucl Med.

[19] Loudos G, Tsougos I, Boukis S, Karakatsanis N, Georgoulias P, Theodorou K (2009). A radionuclide dosimetry toolkit based on material-specific Monte Carlo dose kernels. Nucl Med Commun.

[20] Khazaee Moghadam M, Kamali Asl A, Geramifar P, Zaidi H (2016). Evaluating the application of tissue-specific dose kernels instead of water dose kernels in internal dosimetry: a Monte Carlo study. Cancer Biother Radiopharm.

[21] Lee MS, Kim JH, Paeng JC, Kang KW, Jeong JM, Lee DS (2018). Whole-body voxel-based personalized dosimetry: the multiple voxel S-value approach for heterogeneous media with nonuniform activity distributions. J Nucl Med.

[22] Shiri I, Arabi H, Geramifar P, Hajianfar G, Ghafarian P, Rahmim A, et al. Deep-JASC: joint attenuation and scatter correction in whole-body (18)F-FDG PET using a deep residual network. Eur J Nucl Med Mol Imaging. 2020, in press. 10.1007/s00259-020-04852-5. 10.1007/s00259-020-04852-5 32415552

[23] Xiang H, Lim H, Fessler JA, Dewaraja YK. A deep neural network for fast and accurate scatter estimation in quantitative SPECT/CT under challenging scatter conditions. Eur J Nucl Med Mol Imaging. 2020, in press. 10.1007/s00259-020-04840-9. 10.1007/s00259-020-04840-9 PMC7666660 32415551

[24] Dong X, Lei Y, Wang T, Higgins K, Liu T, Curran WJ (2020). Deep learning-based attenuation correction in the absence of structural information for whole-body PET imaging. Phys Med Biol.

[25] Sanaat A, Arabi H, Mainta I, Garibotto V, Zaidi H. Projection-space implementation of deep learning-guided low-dose brain PET imaging improves performance over implementation in image-space. J Nucl Med. 2020, in press. 10.2967/jnumed.119.239327. 10.2967/jnumed.119.239327 PMC7456177 31924718

[26] Zaharchuk G (2019). Next generation research applications for hybrid PET/MR and PET/CT imaging using deep learning. Eur J Nucl Med Mol Imaging.

[27] Shiri I, AmirMozafari Sabet K, Arabi H, Pourkeshavarz M, Teimourian B, Ay MR, et al. Standard SPECT myocardial perfusion estimation from half-time acquisitions using deep convolutional residual neural networks. J Nucl Cardiol. 2020, in press. 10.1007/s12350-020-02119-y. 10.1007/s12350-020-02119-y 32347527

[28] Seo H, Badiei Khuzani M, Vasudevan V, Huang C, Ren H, Xiao R (2020). Machine learning techniques for biomedical image segmentation: an overview of technical aspects and introduction to state-of-art applications. Med Phys.

[29] Mardani M, Dong P, Xing L (2016). Deep-learning based prediction of achievable dose for personalizing inverse treatment planning. Int J Radiat Oncol Biol Phys.

[30] Nguyen D, Long T, Jia X, Lu W, Gu X, Iqbal Z (2019). A feasibility study for predicting optimal radiation therapy dose distributions of prostate cancer patients from patient anatomy using deep learning. Sci Rep.

[31] Ma M, Buyyounouski MK, Vasudevan V, Xing L, Yang Y (2019). Dose distribution prediction in isodose feature-preserving voxelization domain using deep convolutional neural network. Med Phys.

[32] Kearney V, Chan JW, Haaf S, Descovich M, Solberg TD (2018). DoseNet: a volumetric dose prediction algorithm using 3D fully-convolutional neural networks. Phys Med Biol.

[33] Jarrett D, Stride E, Vallis K, Gooding MJ (2019). Applications and limitations of machine learning in radiation oncology. Br J Radiol.

[34] Andreo P (2018). Monte Carlo simulations in radiotherapy dosimetry. Radiat Oncol.

[35] Lee MS, Hwang D, Kim JH, Lee JS (2019). Deep-dose: a voxel dose estimation method using deep convolutional neural network for personalized internal dosimetry. Sci Rep.

[36] Götz TI, Schmidkonz C, Chen S, Al-Baddai S, Kuwert T, Lang E (2020). A deep learning approach to radiation dose estimation. Phys Med Biol.

[37] Peng Z, Fang X, Yan P, Shan H, Liu T, Pei X (2020). A method of rapid quantification of patient-specific organ doses for CT using deep-learning-based multi-organ segmentation and GPU accelerated Monte Carlo dose computing. Med Phys.

[38] Karbalaee M, Shahbazi-Gahrouei D, Tavakoli MB (2017). An approach in radiation therapy treatment planning: a fast, GPU-based Monte Carlo method. J Med Signals Sens.

[39] Jia X, Ziegenhein P, Jiang SB (2014). GPU-based high-performance computing for radiation therapy. Phys Med Biol.

[40] Loevinger R, Pfalzner P, Eisenlohr H, Malo S, Sanielevici A, Nagl J (1969). The IAEA program in medical radiation dosimetry. Ann N Y Acad Sci.

[41] Cristy M (1983). Applying the reciprocal dose principle to heterogeneous phantoms: practical experience from Monte Carlo studies. Phys Med Biol.

[42] Lee C, Lee C, Shah AP, Bolch WE (2006). An assessment of bone marrow and bone endosteum dosimetry methods for photon sources. Phys Med Biol.

[43] Seuntjens J, Strydom W, Shortt K, Podgorsak EB (2005). Dosimetric principles, quantities and units. Radiation oncology physics: a handbook for teachers and students.

[44] Bailey DL, Karp JS, Surti S. Physics and instrumentation in PET. Positron emission tomography: Springer; 2005. p. 13–39.

[45] Li W, Wang G, Fidon L, Ourselin S, Cardoso MJ, Vercauteren T. On the compactness, efficiency, and representation of 3D convolutional networks: brain parcellation as a pretext task. Int Conf Inf Process Med Imaging. 2017:348–60.

[46] Schneider W, Bortfeld T, Schlegel W (2000). Correlation between CT numbers and tissue parameters needed for Monte Carlo simulations of clinical dose distributions. Phys Med Biol.

[47] Waters LS. MCNPX user’s manual. Los Alamos Nat Lab. 2002.

[48] Jan S, Comtat C, Strul D, Santin G, Trebossen R (2005). Monte Carlo simulation for the ECAT EXACT HR+ system using GATE. IEEE Trans Nucl Sci.

[49] Zaker N, Kotasidis F, Garibotto V, Zaidi H (2020). Assessment of lesion detectability in dynamic whole-body PET imaging using compartmental and Patlak parametric mapping. Clin Nucl Med.

[50] Fahrni G, Karakatsanis N, Di Domenicantonio G, Garibotto V, Zaidi H (2019). Does whole-body Patlak 18F-FDG PET imaging improve lesion detectability in clinical oncology?. Eur Radiol.

[51] Hubbell JH, Seltzer SM (1995). Tables of X-ray mass attenuation coefficients and mass energy-absorption coefficients 1 keV to 20 MeV for elements Z= 1 to 92 and 48 additional substances of dosimetric interest.

[52] Stabin MG, Sparks RB, Crowe E (2005). OLINDA/EXM: the second-generation personal computer software for internal dose assessment in nuclear medicine. J Nucl Med.

[53] Chiesa C, Bardiès M, Zaidi H (2019). Voxel-based dosimetry is superior to mean absorbed dose approach for establishing dose-effect relationship in targeted radionuclide therapy. Med Phys.

[54] Howard DM, Kearfott KJ, Wilderman SJ, Dewaraja YK (2011). Comparison of I-131 radioimmunotherapy tumor dosimetry: unit density sphere model versus patient-specific Monte Carlo calculations. Cancer Biother Radiopharm.

[55] Divoli A, Chiavassa S, Ferrer L, Barbet J, Flux GD, Bardies M (2009). Effect of patient morphology on dosimetric calculations for internal irradiation as assessed by comparisons of Monte Carlo versus conventional methodologies. J Nucl Med.

